# Intraosseous versus intravenous vascular access during cardiopulmonary resuscitation for out-of-hospital cardiac arrest: a systematic review and meta-analysis of observational studies

**DOI:** 10.1186/s13049-021-00858-6

**Published:** 2021-03-08

**Authors:** Yu-Lin Hsieh, Meng-Che Wu, Jon Wolfshohl, James d’Etienne, Chien-Hua Huang, Tsung-Chien Lu, Edward Pei-Chuan Huang, Eric H. Chou, Chih-Hung Wang, Wen-Jone Chen

**Affiliations:** 1 Department of Emergency Medicine, Baylor Scott & White All Saints Medical Center, Texas, 1400 8th Ave. Fort Worth, Fort Worth, TX 76104 USA; 2 Department of Internal Medicine, Danbury Hospital, Danbury, CT USA; 3 Department of Emergency Medicine, National Taiwan University Hospital, No.7, Zhongshan S. Rd., Zhongzheng Dist., Taipei City, 100 Taiwan (Republic of China); 4 Department of Emergency Medicine, John Peter Smith Hospital, Fort Worth, TX USA; 5 Department of Emergency Medicine, College of Medicine, National Taiwan University, Taipei, Taiwan; 6 Division of Cardiology, Department of Internal Medicine, National Taiwan University Hospital and National Taiwan University College of Medicine, Taipei, Taiwan

**Keywords:** Cardiac arrest, Cardiopulmonary resuscitation, Intraosseous, Intravenous, Systematic review, Meta-analysis

## Abstract

**Introduction:**

This study is aimed to investigate the association of intraosseous (IO) versus intravenous (IV) route during cardiopulmonary resuscitation (CPR) with outcomes after out-of-hospital cardiac arrest (OHCA).

**Methods:**

We systematically searched PubMed, Embase, Cochrane Library and Web of Science from the database inception through April 2020. Our search strings included designed keywords for two concepts, i.e. vascular access and cardiac arrest. There were no limitations implemented in the search strategy. We selected studies comparing IO versus IV access in neurological or survival outcomes after OHCA. Favourable neurological outcome at hospital discharge was pre-specified as the primary outcome. We pooled the effect estimates in random-effects models and quantified the heterogeneity by the *I*^*2*^ statistics. Time to intervention, defined as time interval from call for emergency medical services to establishing vascular access or administering medications, was hypothesized to be a potential outcome moderator and examined in subgroup analysis with meta-regression.

**Results:**

Nine retrospective observational studies involving 111,746 adult OHCA patients were included. Most studies were rated as high quality according to Newcastle-Ottawa Scale. The pooled results demonstrated no significant association between types of vascular access and the primary outcome (odds ratio [OR], 0.60; 95% confidence interval [CI], 0.27–1.33; *I*^*2*^, 95%). In subgroup analysis, time to intervention was noted to be positively associated with the pooled OR of achieving the primary outcome (OR: 3.95, 95% CI, 1.42–11.02, *p*: 0.02). That is, when the studies not accounting for the variable of “time to intervention” in the statistical analysis were pooled together, the meta-analytic results between IO access and favourable outcomes would be biased toward inverse association. No obvious publication bias was detected by the funnel plot.

**Conclusions:**

The meta-analysis revealed no significant association between types of vascular access and neurological outcomes at hospital discharge among OHCA patients. Time to intervention was identified to be an important outcome moderator in this meta-analysis of observation studies. These results call for the need for future clinical trials to investigate the unbiased effect of IO use on OHCA CPR.

**Supplementary Information:**

The online version contains supplementary material available at 10.1186/s13049-021-00858-6.

## Introduction

In the United States, there are more than 300,000 out-of-hospital cardiac arrests (OHCA) every year, and the survival rate at hospital discharge is around 10% [1]. Resuscitation guidelines [2, 3] suggest epinephrine should be administered in a timely fashion, especially for patients with initial non-shockable rhythms. The alpha-adrenergic effects of epinephrine produce systemic vasoconstriction, increasing coronary and cerebral perfusion pressures, which are believed to be beneficial in facilitating return of spontaneous circulation (ROSC) [4].

Ewy et al. [5] indicated survival was greatest when epinephrine was given very early but decreased rapidly with increasing delay in epinephrine administration. Hansen et al. [6] also revealed that each minute from arrival of emergency medical services (EMS) to epinephrine administration was associated with a 4% decrease in odds of survival for adult OHCA. Hence, obtaining rapid, reliable vascular access during cardiopulmonary resuscitation (CPR) is critical for OHCA. However, although intravenous (IV) administration is typically recommended [2, 3], establishing IV access is not always fast or practical [7].

Updated guidelines by American Heart Association consider intraosseous (IO) access an acceptable vascular access [8], while European guidelines suggest considering IO access when IV access is difficult [3]. After proper training, IO access could be established more rapidly than IV access by EMS in the field with a high success rate [7]. Nonetheless, whether this technical advantage could be translated into clinical benefits during CPR has not yet been proven by clinical trials. To better understand the clinical effects of vascular access used during cardiac arrest, we conducted this systematic review and meta-analysis to determine if the route of medication administration was associated with neurological or survival outcomes of OHCA.

## Methods

This systematic review and meta-analysis were performed in accordance with the guidelines of PRISMA (Preferred Reporting Items for Systematic reviews and Meta-Analyses) [9] and MOOSE (Meta-analysis of Observational Studies in Epidemiology) [10]. The study protocol was registered with PROSPERO. The registration number is CRD42020179894.

### Data sources and searches

Two investigators (YLH and MCW) independently searched the databases, including PubMed, Embase, Cochrane Library and Web of Science, from the database inception through April 2020. Our search strings included designed keywords for two concepts, i.e. vascular access and cardiac arrest (Additional file 1). We set no restrictions on publication year or language. To ensure completeness, we also screened relevant review articles and meta-analyses for references not captured by our search strategy.

### Study selection

Two investigators (JW and JE) independently scanned the titles and abstracts of all retrieved articles to determine whether the articles were pertinent to this review. We used the following prespecified inclusion criteria: (a) population included patients with OHCA, (b) comparison between IO and IV access for medications administration during CPR, (c) outcomes included survival or neurological results, and (d) study designs included randomized controlled trials, quasi-randomized controlled trials, and observational studies (cohort studies and case-control studies). Case series, reviews, editorials, comments and studies on non-human subjects were not included. We excluded studies that included trauma patients. Full-text articles were retrieved if either of the investigators considered the abstract potentially suitable. After retrieving the full reports of potentially relevant studies, two investigators (JW and JE) independently assessed each study’s eligibility on the basis of the inclusion criteria. Differences of opinion regarding study eligibility were settled by consultation with another investigators (TCL and EPCH).

### Data extraction and quality assessment

In this review, favorable neurological outcome at hospital discharge was pre-specified as the primary outcome. Short-term survival and survival at hospital discharge were the secondary outcomes. Since the definitions for short-term survival were various, we abstracted those outcome data for which the timing was closest to hospital admission.

Two investigators (CHH and EHC) independently extracted qualitative and quantitative data according to a predesigned spreadsheet (Excel [Microsoft]) that was pilot-tested beforehand; a third investigator (WJC) adjudicated discordant assessments. Data were extracted for author information, publication year, study design, study setting, patient number, patient characteristics, selection method for intervention and comparator, definitions of outcomes, unadjusted/adjusted effect estimates and their corresponding 95% confidence intervals (CIs). The patient number was extracted on an intention-to-treat basis. If more than one adjusted effect estimates were reported in a single study, we selected the representative effect estimate according to the following hierarchy of priority: (a) effect estimates derived from multivariable statistical model, (b) effect estimates specifically adjusted for time to intervention, (c) comparison between patients categorized by first attempted vascular access, (d) effect estimates comparing IO versus IV access for active medications, and (e) effect estimates calculated with the largest patient number. If an adjusted effect estimate was not available, an unadjusted one was recorded or calculated manually for analysis. If included studies provided additional effect estimates of IO versus IV access for patients with shockable rhythms, these estimates would also be extracted.

Without blinding to study authors or journals, two investigators (CHH and EHC) independently assessed the study quality using the Newcastle-Ottawa Scale, which rates the quality of observational studies in a standardized and structured format [11]. Conflicts were resolved either by consensus or by the adjudicator (WJC).

### Data synthesis and analysis

Odds ratio (OR) was selected as the primary effect estimate for data synthesis. Since the proportion of patients recovering favorable neurological outcome in OHCA was relatively small, all other measures of effect estimates, such as risk ratio (RR), were assumed to approximate the OR and pooled in meta-analyses if OR could not be obtained. Weighted means of the ORs, with their associated 95% CIs, were calculated in random-effects models (DerSimonian-Laird method [12]) with the Knapp and Hartung adjustment [13]. Heterogeneity was estimated using restricted maximum-likelihood estimation [14] and quantified by the *I*^*2*^ statistics, with *I*^*2*^ > 50% deemed as the presence of significant heterogeneity [15, 16].

To examine heterogeneity, we performed subgroup analysis based on predefined moderator variables, including study year and location, selection method for intervention and comparator (defined as vascular access of first attempted versus actual access for medications administration), EMS response time (defined as time interval from call to EMS arrival), time to intervention (defined as time interval from EMS call to establishing vascular access or administering medications) adjusted in analysis, and type of effect estimates pooled in meta-analysis (adjusted versus unadjusted). In the subgroup analysis, the effect sizes stratified on the same moderator were re-estimated and compared in mixed-effects meta-regression analysis to examine the impact of each moderator on pooled ORs. Additional effect estimates for patients with shockable rhythms were also synthesized in the subgroup analysis. Finally, the presence of publication bias was examined using funnel plots.

The *metafor* package and *rma* function were used to perform meta-analysis and meta-regression in the R 3.6.3 software (R Foundation for Statistical Computing, Vienna, Austria). In the statistical testing, a 2-sided *p* < 0.05 was considered statistically significant.

## Results

### Search results and study characteristics

**Fig. 1:**
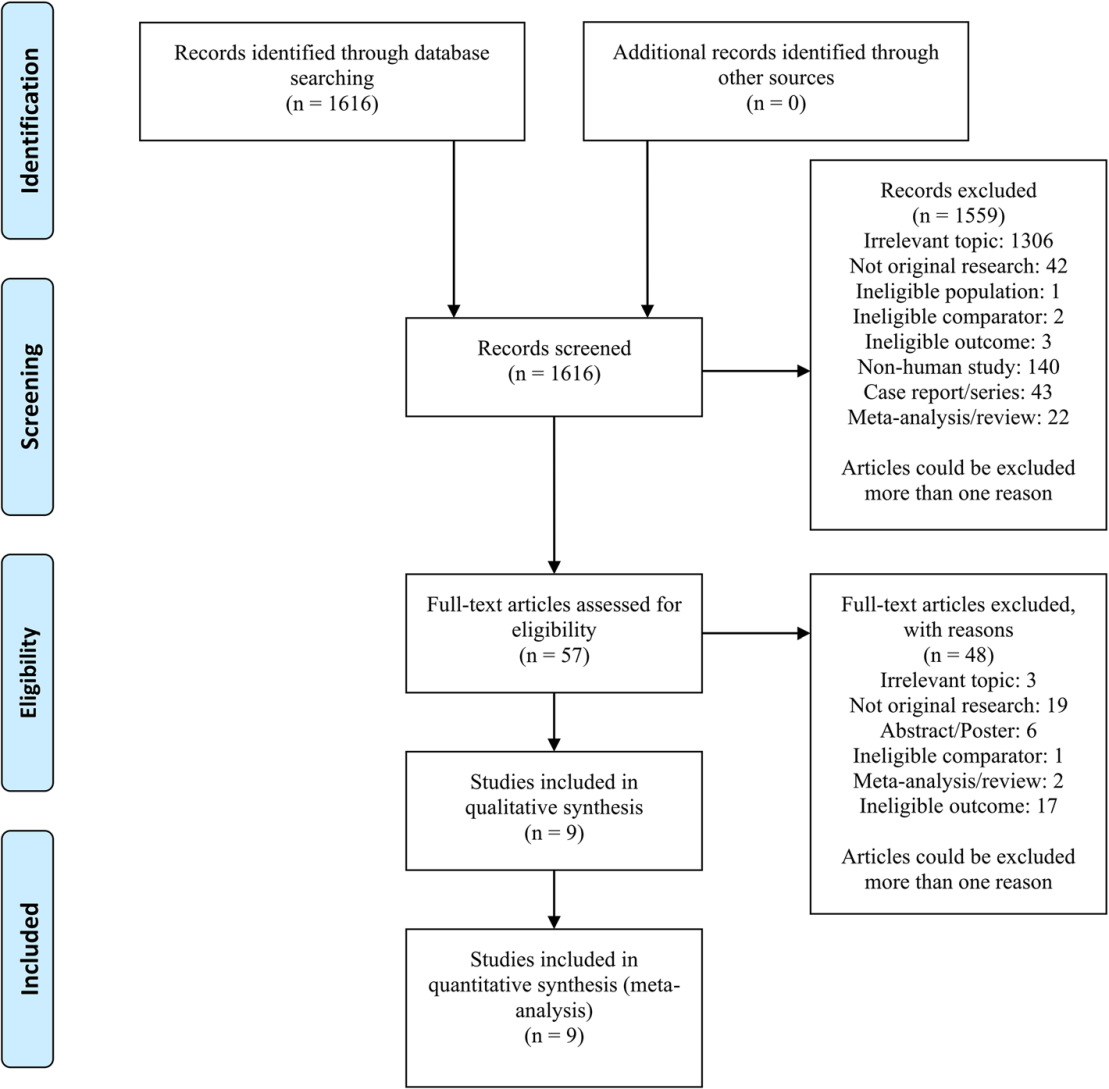
Literature search and study selection flow diagram

After a systematic literature search, we included nine studies [17–25] involving 111,746 adult OHCA patients (Fig. 1; Table 1). All studies were retrospective observational studies and included patients between the years 2007 and 2017, most of which were conducted in North America [17–21, 23, 25].

**Table 1:** Characteristics of the studies identified in the systematic review

First author, publication year	Study period/ region	Patient number	Main population characteristics						Selection of intervention and comparator	EMS^e^ response time, mean or median (min)	Time to intervention adjusted in analysis
			Inclusion criteria	Age, mean or median	Male, %	Witnessed arrest, %	Bystander CPR,^b^ %	Shockable rhythms, %			
Clemency et al., 2017 [17]	2013–2015/ USA	1340	Adult OHCA^a^	62	64	49	27	15	First attempted IO^c^ and IV^d^ access	NA	No
Feinstein et al., 2017 [18]	2012–2014/ USA	1800	Adult non-traumatic OHCA	64	63	57	69	28	Actual IO and IV access for drug use	5.9	Yes (time to establishment of vascular access adjusted)
Kawano et al., 2018 [19]	2007–2009/ USA & Canada	13,155	Adult non-traumatic OHCA	68	66	52	38	26	First attempted IO and IV access	5.5	No
Mody et al., 2019 [20]	2011–2015/ USA & Canada	19,731	Adult non-traumatic OHCA	68	65	24	46	22	First attempted IO and IV access	5.4	Yes (time to medications administration adjusted)
Nguyen et al., 2019 [21]	2013–2017/ USA	795	Adult non-traumatic OHCA	65	62	NA	NA	21	First attempted IO and IV access	12.9	No
Baert et al., 2020 [22]	2011–2017/ France	28,856	Adult non-traumatic OHCA	67	70	73	50	13	Actual IO and IV access for drug use	10	Yes (time to medications administration adjusted)
Daya et al., 2020 [23]	2012–2015/ USA & Canada	3019	Adult non-traumatic OHCA	63	80	70	57	100	Actual IO and IV access for drug use	5.7	Yes (time to establishment of vascular access adjusted)
Nolan et al., 2020 [24]	2014–2017/ UK	7317	Adult non-traumatic OHCA	70	65	61	59	18	Actual IO and IV access for drug use	7.4	No
Zhang et al., 2020 [25]	2011–2015/ USA & Canada	35,733	Adult non-traumatic OHCA	66	66	48	44	22	First attempted IO and IV access	5.1	Yes (time to establishment of vascular access adjusted)

^a^*OHCA* out-of-hospital cardiac arrest

^b^*CPR* cardiopulmonary resuscitation

^c^*IO* intraosseous

^d^*IV* intravenous

^e^*EMS* emergency medical service

The patient demographic data were similar across included studies, except the study by Daya et al. [23], which exclusively enrolled patients with shock-refractory OHCA. Because all studies were retrospective, studied patients were not randomized to IO or IV access; instead, patients were categorized according to the first attempted vascular access [17, 19–21, 25] or the actual access through which the medications were administered [18, 22–24]. Because multiple EMS agencies were involved across the included studies, the policies regarding IO use were various: some recommended IO use at the discretion of healthcare providers [17, 23], some recommended IO use after failed attempt in establishing IV access [18, 21, 23, 24], and others recommended IO as the primary attempt [23].

Most studies used multivariable analysis to adjust the confounding effects of multiple variables, except two studies [17, 21]. Despite using multivariable analysis, Daya et al. [23] and Nolan et al. [24] mainly studied the interaction between vascular access and different medications administered, without providing the effect estimates directly comparing IO versus IV access for the whole cohort. Daya et al. [23] only provided the adjusted RRs comparing IO versus IV access in the placebo group, for whom the epinephrine or other medications were still administered through IO or IV access, and accordingly, these RRs were abstracted. In contrast, for the Nolan et al. [24] study, only the unadjusted OR calculated manually from the provided data in the group receiving adrenaline was abstracted. Five studies [18, 20, 22, 23, 25] provided the effect estimates adjusted by time to intervention. Three studies [19, 20, 25] provided additional effect estimates for a subgroup of patients with shockable rhythms, which would be pooled with the effect estimates of the total cohort provided by Daya et al. [23].

Most studies [19, 20, 23–25] used Modified Rankin Score≦3 to define the favorable neurological outcome at hospital discharge whereas Baert et al. [22] defined it as Cerebral Performance Category Score≦2. Short-term survival was defined differently across the included studies, including any ROSC [19, 21], ROSC before arrival at emergency department (ED) [25], ROSC at ED arrival [17, 20, 24], and survival to hospital admission [18, 22, 23].

Most studies achieved similarly high Newcastle-Ottawa Scale scores (Additional file 2), except two studies [17, 21] which only provided unadjusted effect estimates for short-term survival.

### Quantitative synthesis

All studies provided ORs as the effect estimates, except that Daya et al. [23] reported RRs. For the primary outcome (Table 2), 6 studies [19, 20, 22–25] reported the neurological outcome at hospital discharge; for secondary outcomes (Table 3), 9 [17–25] and 7 [18–20, 22–25] studies reported short-term survival and survival at hospital discharge, respectively.

**Table 2:** Results of the summary effect estimates for the primary outcome

Analysis	Outcome or moderator variables	Number of included studies	Odds ratio (95% confidence interval)	*I*^*2*^, %	*p* for mixed-effects meta-regression analysis
Main analysis	Favorable neurological outcome at hospital discharge	6 [19, 20, 22–25]	0.60 (0.27–1.33)	95	NA
Subgroup analysis					
Study period	Before 2010	1 [19]	0.22 (0.11–0.42)	0	0.19
	After 2010	5 [20, 22–25]	0.73 (0.33–1.63)	94	
Study region	North America	4 [19, 20, 23, 25]	0.60 (0.20–1.79)	94	> 0.99
	Europe	2 [22, 24]	0.57 (9.94E-06-32,578.24)	94	
Selection of vascular access	First attempted vascular access	3 [19, 20, 25]	0.49 (0.09–2.66)	94	0.56
	Actual vascular access	3 [22–24]	0.73 (0.07–7.22)	95	
EMS response time	Longer than 10 mins	1 [22]	1.30 (0.97–1.73)	0	0.29
	Shorter than 10 mins	5 [19, 20, 23–25]	0.51 (0.21–1.26)	94	
Time to intervention	Timing adjusted in analysis	4 [20, 22, 23, 25]	0.89 (0.48–1.66)	89	0.02
	Timing not adjusted in analysis	2 [19, 24]	0.22 (0.17–0.30)	0	
Type of effect estimates pooled in analysis	Adjusted effect estimates	5 [19, 20, 22, 23, 25]	0.71 (0.30–1.65)	95	0.25
	Unadjusted effect estimates	1 [24]	0.23 (0.11–0.50)	0	
Initial arrest rhythm	Shockable rhythms	4 [19, 20, 23, 25]	0.64 (0.27–1.53)	90	NA

**Table 3:** Results of the summary effect estimates for the secondary outcomes

Analysis	Outcome or moderator variables	Number of included studies	Odds ratio (95% confidence interval)	*I*^*2*^, %	*p* for mixed-effects meta-regression analysis
Main analysis	Short-term survival	9 [17–25]	0.71 (0.59–0.85)	86	NA
Subgroup analysis					
Study period	Before 2010	1 [19]	0.58 (0.48–0.70)	0	0.39
	After 2010	8 [17, 18, 20–25]	0.73 (0.60–0.89)	87	
Study region	North America	7 [17–21, 23, 25]	0.74 (0.59–0.94)	88	0.36
	Europe	2 [22, 24]	0.62 (0.23–1.69)	40	
Selection of vascular access	First attempted vascular access	5 [17, 19–21, 25]	0.70 (0.50–0.98)	90	0.90
	Actual vascular access	4 [18, 22–24]	0.72 (0.49–1.04)	82	
EMS response time	Longer than 10 mins	2 [21, 22]	0.59 (0.09–4.02)	67	0.24
	Shorter than 10 mins	7 [17–20, 23–25]	0.75 (0.61–0.92)	87	
Time to intervention	Timing adjusted in analysis	5 [18, 20, 22, 23, 25]	0.77 (0.64–0.92)	76	0.25
	Timing not adjusted in analysis	4 [17, 19, 21, 24]	0.64 (0.39–1.05)	85	
Type of effect estimates pooled in analysis	Adjusted effect estimates	6 [18–20, 22, 23, 25]	0.73 (0.61–0.88)	82	0.55
	Unadjusted effect estimates	3 [17, 21, 24]	0.66 (0.25–1.70)	88	
Initial arrest rhythm	Shockable rhythms	4 [19, 20, 23, 25]	0.72 (0.48–1.09)	80	NA
Main analysis	Survival at hospital discharge	7 [18–20, 22–25]	0.66 (0.42–1.04)	89	NA
Subgroup analysis					
Study period	Before 2010	1 [19]	0.40 (0.26–0.61)	0	0.31
	After 2010	6 [18, 20, 22–25]	0.72 (0.44–1.19)	88	
Study region	North America	5 [18–20, 23, 25]	0.75 (0.47–1.20)	86	0.30
	Europe	2 [22, 24]	0.45 (0.0003–521.08)	87	
Selection of vascular access	First attempted vascular access	3 [19, 20, 25]	0.64 (0.24–1.69)	90	0.84
	Actual vascular access	4 [18, 22–24]	0.68 (0.25–1.85)	87	
EMS response time	Longer than 10 mins	1 [22]	0.76 (0.47–1.23)	0	0.79
	Shorter than 10 mins	6 [18–20, 23–25]	0.64 (0.37–1.13)	92	
Time to intervention	Timing adjusted in analysis	5 [18, 20, 22, 23, 25]	0.83 (0.64–1.08)	66	0.01
	Timing not adjusted in analysis	2 [19, 24]	0.34 (0.02–5.91)	31	
Type of effect estimates pooled in analysis	Adjusted effect estimates	6 [18–20, 22, 23, 25]	0.75 (0.53–1.07)	81	0.06
	Unadjusted effect estimates	1 [24]	0.25 (0.13–0.47)	0	
Initial arrest rhythm	Shockable rhythms	4 [19, 20, 23, 25]	0.78 (0.44–1.41)	80	NA

**Fig. 2:**
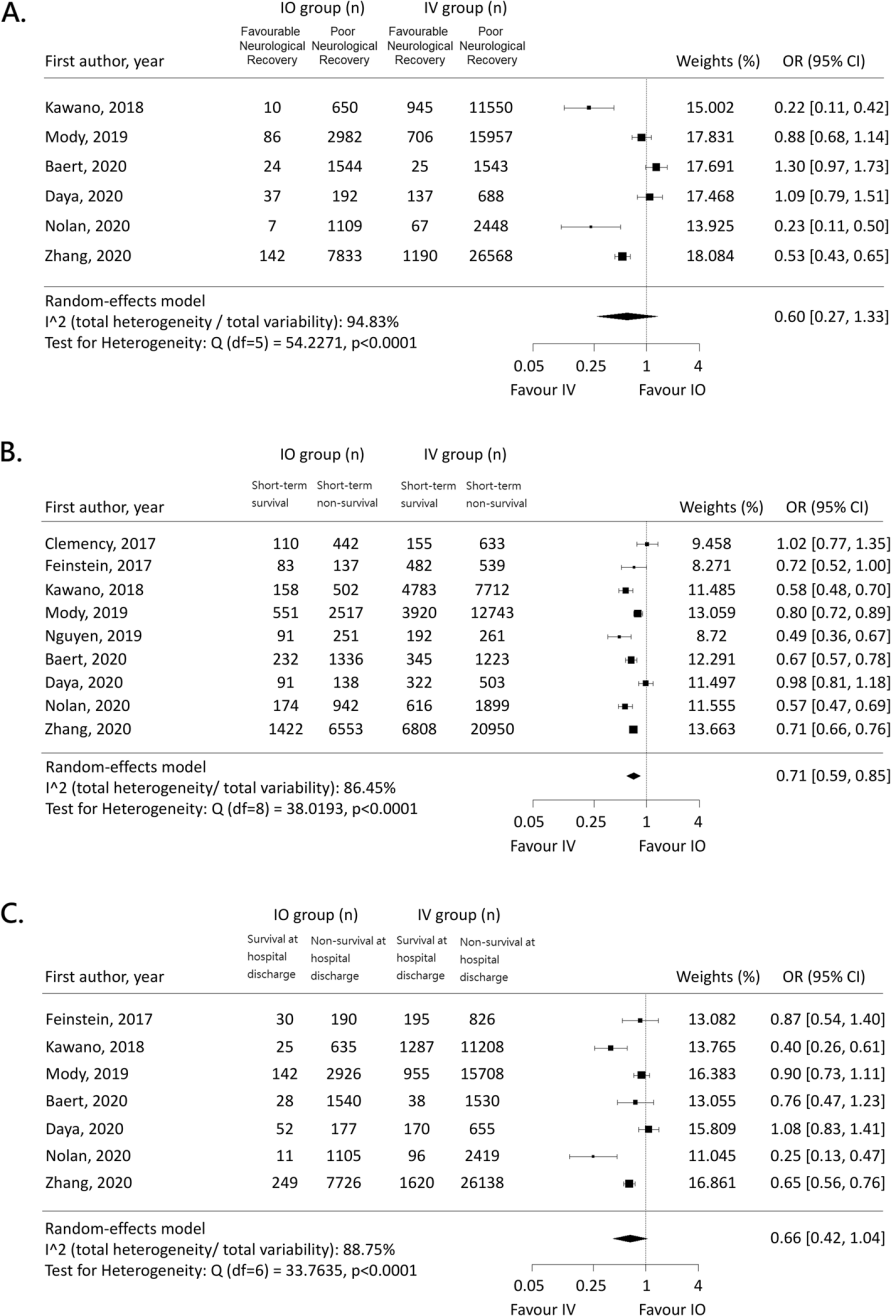
**a** Forest plot for pooled odds ratio of favorable neurological outcome at hospital discharge; **b** Forest plot for pooled odds ratio of short-term survival; **c** Forest plot for pooled odds ratio of survival at hospital discharge. IO, intraosseous; IV, intravascular; OR, odds ratio

For the primary outcome, the pooled results demonstrated no significant association between vascular access type and neurological outcome (OR, 0.60; 95% CI, 0.27–1.33; *I*^*2*^, 95%; Fig. 2a; Table 2). For the secondary outcomes, IO access was inversely associated with short-term survival (OR, 0.71; 95% CI, 0.59–0.85; *I*^*2*^, 86%; Table 3; Fig. 2b) while not significantly associated with survival at hospital discharge (OR, 0.66; 95% CI, 0.42–1.04; *I*^*2*^, 89%; Table 3; Fig. 2c).

Significant heterogeneity was observed across the main analyses. In the subgroup analyses (Tables 2 and 3), the meta-regression analysis suggested that time to intervention may be a significant outcome moderator. For the primary outcome, when the studies not adjusting time to intervention were pooled, the heterogeneity decreased and IO access was inversely associated with favorable neurological outcome (OR, 0.22; 95% CI, 0.17–0.30; *I*^*2*^, 0%; Table 2). The meta-regression analysis indicated that time to intervention was positively associated with the pooled OR of favorable neurological outcome (OR: 3.95, 95% CI, 1.42–11.02, *p*: 0.02; Table 2).

For the secondary outcomes, when studies were stratified on time to intervention, the heterogeneity of the pooled ORs decreased for both subgroups despite that the associations between vascular access and survival at hospital discharge remained non-significant (Table 3). The meta-regression analysis also indicated that time to intervention was positively associated with the pooled OR of survival at hospital discharge (OR: 2.37, 95% CI, 1.62–3.45, *p*: 0.01; Table 3).

For the primary outcome, the funnel plot did not reveal obvious publication bias, according to mixed-effects meta-regression model (Additional file 3).

## Discussion

### Main findings

This systematic review identified nine retrospective observational studies comparing IO versus IV access in adult OHCA patients. The meta-analytic results revealed no significant association between types of vascular access and neurological or survival outcomes at hospital discharge. Substantial heterogeneity was noted across these pooled effect estimates. Subgroup analysis with meta-regression indicated that “time to intervention” was a significant outcome moderator. That is, when the studies not accounting for the variable of “time to intervention” in the statistical analysis were pooled together, the meta-analytic results between IO access and favourable outcomes would be biased toward inverse association.

### Comparisons with previous studies

Two previous meta-analyses [26, 27] indicated that IO access was associated with worse OHCA outcomes, compared with IV access. In Morales-Cané et al. study [26], the synthesized OR from three studies [18–20] indicated significant association between IO access and lower survival at hospital discharge (*I*^*2*^, 30%). Subsequently, after adding the study by Zhang et al. [25] to the previous results [26], Granfeldt et al. [27] indicated that IO access was not only associated with lower survival (*I*^*2*^, 71%) but also worse neurological outcome at hospital discharge (*I*^*2*^, 89%), though the heterogeneity of this expanded meta-analysis increased substantially. Interestingly, it was the latest Zhang et al. study [25] that predominantly influenced the pooled results by Granfeldt et al. [27], accounting for approximately 60 and 40% of the total weighting in the fixed-effects and random-effects model, respectively. Nonetheless, in the Zhang et al. [25] study, approximately 47% of patients were excluded from the final analysis, resulting in a highly-selected cohort, thereby probably magnifying the statistical association between vascular access type and outcomes.

Compared to these previous studies [26, 27], our study further included results from secondary analyses [23, 24] of two large clinical trials [28, 29]. As a result, the weighting for each study in our random-effects model was more comparable across all pooled outcomes, abating the dominant influence exerted by the Zhang et al. [25] study and shifting the ORs from favoring IV access toward being inconclusive. Nonetheless, our heterogeneity was still substantially high, as noted by Granfeldt et al. [27], necessitating a measured interpretation of our analysis and further investigation of the sources of the heterogeneity.

### Interpretation of current analyses

Despite that most of the included studies used multiple statistical methods to account for confounding factors and were rated as high quality on Newcastle-Ottawa Scale, the inherent limitations of the retrospective observational study design may still limit the interpretation of current meta-analytic results, indicating the need for a high-quality clinical trial.

First, the selection method in vascular access, i.e. first attempted versus actual access, may lead to confounding by indication. Successful IV cannulation can be challenging during CPR because of compromised peripheral vasculature. Depending on the EMS policy, IO access may be used as the last resort for medications administration [18, 21, 23, 24] because of its high success rate during CPR [7]. Characteristics of patients for whom IV access was difficult may be different from those for whom IV access is more easily established, such as higher proportions of obesity in the former, which by itself may be associated with worse outcomes [30, 31].

Second, not accounting for time to intervention may introduce resuscitation time bias [32], i.e. longer time to intervention, hence longer CPR time, may itself be inversely associated with outcomes, irrespective of which type of vascular access was used. Indeed, our meta-regression analysis indicates that when time to intervention is adjusted, the pooled ORs may be directed toward favoring IO access, compared with those pooled with studies not accounting for time to intervention. To better deal with resuscitation time bias, risk set matching [32], such as time-dependent propensity score matching [33], may be employed, however such an analysis would necessitate a larger number of cases than currently exists in the OHCA literature. Moreover, establishing IO or IV access itself does not benefit patients but rather the medications administered through the established access. Therefore, compared with time to establishing vascular access, adjusting time to administering medications through access of interests may be more relevant to the comparison between IO versus IV access; nonetheless, only two studies [20, 22] had considered timing of medication administration in their analysis.

### Future implications

Because of the above-mentioned risks of bias, the meta-analytic results should be applied cautiously in clinical practice. Previous studies [5, 6] demonstrated the chances of achieving favorable neurological or survival outcome rapidly decreased with delay in adrenaline administration. In a swine model, Zuercher et al. [34] showed that early IO adrenaline administration was superior to delayed IV adrenaline injection in achieving 24-h survival. Therefore, despite that the meta-analytic results demonstrated inconclusive or non-significant association between either vascular access with OHCA outcomes, emergency care providers should still take into account the influence of delay in medication administration on outcomes when they attempt to establish vascular access, especially for patients with non-shockable rhythms [35]. It should especially be emphasized that no relevant clinical outcome-directed studies were identified in current review for pediatric patients, whose vascular access was difficult to be obtained during CPR. The trade-off between the technical advantage by IO access in facilitating early medications administration and the potentially reduced medications effects by IO route should be further examined in future clinical trials.

### Study limitations

There are some limitations in our study. First, we did not have enough information to include the site of IO insertion in the subgroup analysis. Humeral IO access of adrenaline administration has been shown to reach higher maximum serum concentration in a shorter time, compared with tibial IO access, thereby leading to higher chances of survival [36]. It was possible that differences in IO site selection could partly explain the substantial heterogeneity. Second, for each study, we only abstracted one representative effect estimate for synthesis in the meta-analysis, without testing combinations of all potentially available effect estimates. Nonetheless, we thought that a hypothesis-driven meta-analysis with a predefined algorithm for abstraction could probably avoid type I error. Finally, despite that all included studies used accepted assessment systems to categorize neurological outcomes, these outcomes were retrospectively assessed in most studies, which may introduce misclassification bias, leading to non-significant results.

## Conclusions

The meta-analysis revealed no significant association between types of vascular access and neurological or survival outcomes at hospital discharge among OHCA patients. Time to intervention was identified to be an important outcome moderator in this meta-analysis of observational studies. These results call for the need for future clinical trials to investigate the unbiased effect of IO use on OHCA CPR.

## Supplementary Information

**Additional file 1.** Search strategy for each database.

**Additional file 2.** Newcastle-Ottawa quality assessment scale for cohort studies.

**Additional file 3.** Funnel plot for favorable neurological outcome at hospital discharge, according to mixed-effects meta-regression model.

## Data Availability

The datasets used and/or analysed during the current study are available from the corresponding author on reasonable request.

## Abbreviations

CI: Confidence interval
CPR: Cardiopulmonary resuscitation
ED: Emergency department
EMS: Emergency medical services
IO: Intraosseous
IV: Intravenous
OHCA: Out-of-hospital cardiac arrests
OR: Odds ratio
ROSC: Return of spontaneous circulation
RR: Risk ratio

## References

[1] Benjamin EJ, Muntner P, Alonso A, Bittencourt MS, Callaway CW, Carson AP, Chamberlain AM, Chang AR, Cheng S, Das SR, Delling FN, Djousse L, MSV E, Ferguson JF, Fornage M, Jordan LC, Khan SS, Kissela BM, Knutson KL, Kwan TW, Lackland DT, Lewis TT, Lichtman JH, Longenecker CT, Loop MS, Lutsey PL, Martin SS, Matsushita K, Moran AE, Mussolino ME, O'Flaherty M, Pandey A, Perak AM, Rosamond WD, Roth GA, UKA S, Satou GM, Schroeder EB, Shah SH, Spartano NL, Stokes A, Tirschwell DL, Tsao CW, Turakhia MP, LB VW, Wilkins JT, Wong SS, Virani SS, American Heart Association Council on Epidemiology and Prevention Statistics Committee and Stroke Statistics Subcommittee (2019). Heart disease and stroke statistics-2019 update: a report from the american heart association. Circulation.

[2] Link MS, Berkow LC, Kudenchuk PJ, Halperin HR, Hess EP, Moitra VK, Neumar RW, O'Neil BJ, Paxton JH, Silvers SM, White RD, Yannopoulos D, Donnino MW (2015). Part 7: adult advanced cardiovascular life support: 2015 American Heart Association guidelines update for cardiopulmonary resuscitation and emergency cardiovascular care. Circulation.

[3] Soar J, Nolan JP, Böttiger BW, Perkins GD, Lott C, Carli P, Pellis T, Sandroni C, Skrifvars MB, Smith GB, Sunde K, Deakin CD, Adult advanced life support section Collaborators (2015). European resuscitation council guidelines for resuscitation 2015: section 3. Adult advanced life support. Resuscitation.

[4] Bar-Joseph G, Weinberger T, Ben-Haim S (2000). Response to repeated equal doses of epinephrine during cardiopulmonary resuscitation in dogs. Ann Emerg Med.

[5] Ewy GA, Bobrow BJ, Chikani V, Sanders AB, Otto CW, Spaite DW, Kern KB (2015). The time dependent association of adrenaline administration and survival from out-of-hospital cardiac arrest. Resuscitation.

[6] Hansen M, Schmicker RH, Newgard CD, Grunau B, Scheuermeyer F, Cheskes S, Vithalani V, Alnaji F, Rea T, Idris AH, Herren H, Hutchison J, Austin M, Egan D, Daya M (2018). Resuscitation outcomes consortium investigators. Time to epinephrine administration and survival from Nonshockable out-of-hospital cardiac arrest among children and adults. Circulation.

[7] Reades R, Studnek JR, Vandeventer S, Garrett J (2011). Intraosseous versus intravenous vascular access during out-of-hospital cardiac arrest: a randomized controlled trial. Ann Emerg Med.

[8] Panchal AR, Berg KM, Kudenchuk PJ, Del Rios M, Hirsch KG, Link MS, Kurz MC, Chan PS, Cabañas JG, Morley PT, Hazinski MF, Donnino MW (2018). 2018 American Heart Association focused update on advanced cardiovascular life support use of antiarrhythmic drugs during and immediately after cardiac arrest: an update to the American Heart Association guidelines for cardiopulmonary resuscitation and emergency cardiovascular care. Circulation.

[9] Moher D, Liberati A, Tetzlaff J, Altman DG, PRISMA Group (2009). Preferred reporting items for systematic reviews and meta-analyses: the PRISMA statement. Ann Intern Med.

[10] Stroup DF, Berlin JA, Morton SC, Olkin I, Williamson GD, Rennie D, Moher D, Becker BJ, Sipe TA, Thacker SB (2000). Meta-analysis of observational studies in epidemiology: a proposal for reporting. Meta-analysis of observational studies in epidemiology (MOOSE) group. JAMA.

[11] Wells GA, Shea B, O'Connell D, Peterson J, Welch V, Losos M, Tugwell P (2008). The Newcastle-Ottawa scale (NOS) for assessing the quality of nonrandomised studies in meta-analyses.

[12] DerSimonian R, Laird N (1986). Meta-analysis in clinical trials. Control Clin Trials.

[13] Knapp G, Hartung J (2003). Improved tests for a random effects meta-regression with a single covariate. Stat Med.

[14] Viechtbauer W (2005). Bias and efficiency of meta-analytic variance estimators in the random-effects model. J Educ Behav Stat.

[15] Higgins JP, Thompson SG, Deeks JJ, Altman DG (2003). Measuring inconsistency in meta-analyses. BMJ.

[16] Ioannidis JP, Patsopoulos NA, Evangelou E, Patsopoulos N, Evangelou E (2007). Uncertainty in heterogeneity estimates in meta-analyses. BMJ.

[17] Clemency B, Tanaka K, May P, Innes J, Zagroba S, Blaszak J, Hostler D, Cooney D, McGee K, Lindstrom H (2017). Intravenous vs. intraosseous access and return of spontaneous circulation during out of hospital cardiac arrest. Am J Emerg Med.

[18] Feinstein BA, Stubbs BA, Rea T, Kudenchuk PJ (2017). Intraosseous compared to intravenous drug resuscitation in out-of-hospital cardiac arrest. Resuscitation.

[19] Kawano T, Grunau B, Scheuermeyer FX, Gibo K, Fordyce CB, Lin S, Stenstrom R, Schlamp R, Jenneson S, Christenson J (2018). Intraosseous vascular access is associated with lower survival and neurologic recovery among patients with out-of-hospital cardiac arrest. Ann Emerg Med.

[20] Mody P, Brown SP, Kudenchuk PJ, Chan PS, Khera R, Ayers C, Pandey A, Kern KB, de Lemos JA, Link MS, Idris AH (2019). Intraosseous versus intravenous access in patients with out-of-hospital cardiac arrest: insights from the resuscitation outcomes consortium continuous chest compression trial. Resuscitation.

[21] Nguyen L, Suarez S, Daniels J, Sanchez C, Landry K, Redfield C (2019). Effect of intravenous versus Intraosseous access in Prehospital cardiac arrest. Air Med J.

[22] Baert V, Vilhelm C, Escutnaire J, Nave S, Hugenschmitt D, Chouihed T, Tazarourte K, Javaudin F, Wiel E, El Khoury C, Hubert H (2020). GR-RéAC. Intraosseous versus peripheral intravenous access during out-of-hospital cardiac arrest: a comparison of 30-day survival and neurological outcome in the French national registry. Cardiovasc Drugs Ther.

[23] Daya MR, Leroux BG, Dorian P, Rea TD, Newgard CD, Morrison LJ, Lupton JR, Menegazzi JJ, Ornato JP, Sopko G, Christenson J, Idris A, Mody P, Vilke GM, Herdeman C, Barbic D, Kudenchuk PJ (2020). Resuscitation outcomes consortium investigators: survival after intravenous versus Intraosseous Amiodarone, Lidocaine, or placebo in out-of-hospital shock-refractory cardiac arrest. Circulation.

[24] Nolan JP, Deakin CD, Ji C, Gates S, Rosser A, Lall R, Perkins GD (2020). Intraosseous versus intravenous administration of adrenaline in patients with out-of-hospital cardiac arrest: a secondary analysis of the PARAMEDIC2 placebo-controlled trial. Intensive Care Med.

[25] Zhang Y, Zhu J, Liu Z, Gu L, Zhang W, Zhan H, Hu C, Liao J, Xiong Y, Idris AH (2020). Intravenous versus intraosseous adrenaline administration in out-of-hospital cardiac arrest: a retrospective cohort study. Resuscitation.

[26] Morales-Cané I, Valverde-León MDR, Rodríguez-Borrego MA, López-Soto PJ (2020). Intraosseous access in adults in cardiac arrest: a systematic review and meta-analysis. Emergencias.

[27] Granfeldt A, Avis SR, Lind PC, Holmberg MJ, Kleinman M, Maconochie I, Hsu CH, Fernanda de Almeida M, Wang TL, Neumar RW, Andersen LW (2020). Intravenous vs. intraosseous administration of drugs during cardiac arrest: a systematic review. Resuscitation.

[28] Kudenchuk PJ, Brown SP, Daya M, Rea T, Nichol G, Morrison LJ, Leroux B, Vaillancourt C, Wittwer L, Callaway CW, Christenson J, Egan D, Ornato JP, Weisfeldt ML, Stiell IG, Idris AH, Aufderheide TP, Dunford JV, Colella MR, Vilke GM, Brienza AM, Desvigne-Nickens P, Gray PC, Gray R, Seals N, Straight R, Dorian P (2016). Resuscitation outcomes consortium investigators: Amiodarone, Lidocaine, or placebo in out-of-hospital cardiac arrest. N Engl J Med.

[29] Perkins GD, Ji C, Deakin CD, Quinn T, Nolan JP, Scomparin C, Regan S, Long J, Slowther A, Pocock H, Black JJM, Moore F, Fothergill RT, Rees N, O'Shea L, Docherty M, Gunson I, Han K, Charlton K, Finn J, Petrou S, Stallard N, Gates S, Lall R (2018). PARAMEDIC2 collaborators: a randomized trial of epinephrine in out-of-hospital cardiac arrest. N Engl J Med.

[30] Wang CH, Huang CH, Chang WT, Fu CM, Wang HC, Tsai MS, Yu PH, Wu YW, Ma MH, Chen WJ (2018). Associations between body size and outcomes of adult in-hospital cardiac arrest: a retrospective cohort study. Resuscitation.

[31] Wang CH, Chang WT, Huang CH, Tsai MS, Lu TC, Chou E, Wu YW, Chen WJ (2020). Associations between central obesity and outcomes of adult in-hospital cardiac arrest: a retrospective cohort study. Sci Rep.

[32] Andersen LW, Grossestreuer AV, Donnino MW (2018). “Resuscitation time bias”-a unique challenge for observational cardiac arrest research. Resuscitation.

[33] Andersen LW, Granfeldt A, Callaway CW, Bradley SM, Soar J, Nolan JP, Kurth T, Donnino MW (2017). American Heart Association’s get with the guidelines–resuscitation investigators: association between tracheal intubation during adult in-hospital cardiac arrest and survival. JAMA.

[34] Zuercher M, Kern KB, Indik JH, Loedl M, Hilwig RW, Ummenhofer W, Berg RA, Ewy GA (2011). Epinephrine improves 24-hour survival in a swine model of prolonged ventricular fibrillation demonstrating that early intraosseous is superior to delayed intravenous administration. Anesth Analg.

[35] Ewy GA (2017). Cardiocerebral and cardiopulmonary resuscitation - 2017 update. Acute Med Surg.

[36] Beaumont LD, Baragchizadeh A, Johnson C, Johnson D (2016). Effects of tibial and humerus intraosseous administration of epinephrine in a cardiac arrest swine model. Am J Disaster Med.

