# Supplementary material for: Intraosseous versus intravenous vascular access during cardiopulmonary resuscitation for out-of-hospital cardiac arrest: a systematic review and meta-analysis of observational studies
Source: Scand J Trauma Resusc Emerg Med. 2021 Mar 8;29:44. doi: 10.1186/s13049-021-00858-6 (PMC7938460; doi:10.1186/s13049-021-00858-6)
Supplement: Supplementary file 1 — Additional file 1. Search strategy for each database. [file 13049_2021_858_MOESM1_ESM.docx]

Additional file 1. Search strategy for each database

| Database | Search strategy |
| --- | --- |
| PubMed | ("Infusions, Intraosseous"[Mesh] OR Intraosseous Infusion*[Title/Abstract] OR Intra-Osseous Infusion*[Title/Abstract] OR Intraosseous[Title/Abstract] OR Intra-Osseous[Title/Abstract] OR Intraosseous Infus*[Title/Abstract]) AND ("Heart Arrest"[Mesh] OR heart arrest*[Title/Abstract] OR cardiac arrest*[Title/Abstract] OR cardiopulmonary arrest*[Title/Abstract] OR systole*[Title/Abstract] OR "Death, Sudden, Cardiac"[Mesh] OR sudden cardiac death[Title/Abstract] OR sudden cardiac arrest*[Title/Abstract] OR "Out-of-Hospital Cardiac Arrest"[Mesh] OR Out-of-hospital cardiac arrest*[Title/Abstract] OR out of hospital cardiac arrest*[Title/Abstract] OR "Tachycardia, Ventricular"[Mesh] OR ventricular tachycardia[Title/Abstract] OR "Ventricular Fibrillation"[Mesh] OR ventricular fibrillation*[Title/Abstract] OR pulseless electrical activity[Title/Abstract] OR PEA[Title/Abstract] OR arrest*[Title/Abstract] OR out of hospital[Title/Abstract] OR out-of-hospital [Title/Abstract]) |
| Embase | ('intraosseous drug administration'/exp OR 'intraosseous drug administration') AND [<1966-2020]/py AND ('heart arrest'/exp OR 'heart arrest' OR 'out of hospital cardiac arrest'/exp OR 'out of hospital cardiac arrest' OR 'sudden cardiac death'/exp OR 'sudden cardiac death' OR 'cardiopulmonary arrest'/exp OR 'cardiopulmonary arrest' OR 'experimental heart arrest'/exp OR 'experimental heart arrest' OR 'heart ventricle fibrillation'/exp OR 'heart ventricle fibrillation' OR 'heart ventricle tachycardia'/exp OR 'heart ventricle tachycardia' OR 'pulseless electrical activity' OR 'pulseless electrical activity arrest' OR 'pulseless electric activity' OR 'pulseless ventricular tachycardia' OR intraosseous:ti OR intraosseous:ab OR arrest:ti OR arrest:ab OR 'out of hospital':ti OR asytole:ti OR aystolia:ti OR 'ventricular tachycardia':ti OR 'ventricular tachycardia':ab OR 'pulseless electrical activity':ti) |
| Cochrane Library | 1. MeSH descriptor: [Infusions, Intraosseous] explode all trees 2. (Intraosseous infusions OR intra-osseuous OR infusions OR infusion OR intraosseous):ti,ab,kw 3. MeSH descriptor: [Heart Arrest] explode all trees 4. MeSH descriptor: [Out-of-Hospital Cardiac Arrest] explode all trees 5. (Heart Arrest OR "Out-of-hospital" OR "out of hospital" OR cardiac arrest OR asystole OR asystolia OR asystole arrest OR cardiopulmonary arrest):ti,ab,kw 6. MeSH descriptor: [Death, Sudden, Cardiac] explode all trees 7. "sudden cardiac arrest" OR "sudden cardiac death" 8. MeSH descriptor: [Ventricular Fibrillation] explode all trees 9. ("ventricular tachycardia" OR "ventricular tachycardias" OR "ventricular fibrillation" OR "ventricular fibrillations" OR "pulseless electrical activity" OR "PEA"):ti,ab,kw 10. MeSH descriptor: [Tachycardia, Ventricular] explode all trees 11. #1 OR #2 12. #3 OR #4 OR #5 OR #6 OR #7 OR #8 OR #9 OR #10 13. #11 AND #12 |
| Web of Science | TS=(“cardiac arrest” OR “out-of-hospital cardiac arrest” OR “heart arrest”) AND TS=(intraosseous), Indexes= SCI-EXPANDED, SSCI, A&HCI, ESCI Timespan=All years |
