# Supplementary material for: Intraosseous versus intravenous vascular access during cardiopulmonary resuscitation for out-of-hospital cardiac arrest: a systematic review and meta-analysis of observational studies
Source: Scand J Trauma Resusc Emerg Med. 2021 Mar 8;29:44. doi: 10.1186/s13049-021-00858-6 (PMC7938460; doi:10.1186/s13049-021-00858-6)
Supplement: Supplementary file 2 — Additional file 2. Newcastle-Ottawa quality assessment scale for cohort studies. [file 13049_2021_858_MOESM2_ESM.docx]

Additional file 2. Newcastle-Ottawa quality assessment scale for cohort studies

|  | Selection | | | | Comparability | Outcome | | | Result |
| --- | --- | --- | --- | --- | --- | --- | --- | --- | --- |
| First author, publication year | Representative-ness of the exposed cohort | Selection of the non-exposed cohort | Ascertainment of exposure | Demonstration that outcome of interest was not present at start of study | Comparability of cohorts on the basis of the design or analysis | Assessment of outcome | Was follow-up long enough for outcomes to occur | Adequacy of follow up of cohorts | Total score |
| Clemency et al., 2017 ^17^ | 1 | 1 | 1 | 1 | 0 | 1 | 0 | 1 | 6 |
| Feinstein et al., 2017 ^18^ | 1 | 1 | 1 | 1 | 2 | 1 | 1 | 1 | 9 |
| Kawano et al., 2018 ^19^ | 1 | 1 | 1 | 1 | 2 | 1 | 1 | 1 | 9 |
| Mody et al., 2019 ^20^ | 1 | 1 | 1 | 1 | 2 | 1 | 1 | 1 | 9 |
| Nguyen et al., 2019 ^21^ | 1 | 1 | 1 | 1 | 0 | 1 | 0 | 1 | 6 |
| Baert et al., 2020 ^22^ | 1 | 1 | 1 | 1 | 2 | 1 | 1 | 1 | 9 |
| Daya et al., 2020 ^23^ | 1 | 1 | 1 | 1 | 2 | 1 | 1 | 1 | 9 |
| Nolan et al., 2020 ^24^ | 1 | 1 | 1 | 1 | 2 | 1 | 1 | 1 | 9 |
| Zhang et al., 2020 ^25^ | 1 | 1 | 1 | 1 | 2 | 1 | 1 | 1 | 9 |

A maximum of two points could be allotted in the domain of comparability. For other domains, the maximum point was one point for each category. The maximum of total scores was 9 points.
