# Supplementary material for: Intraosseous versus intravenous vascular access during cardiopulmonary resuscitation for out-of-hospital cardiac arrest: a systematic review and meta-analysis of observational studies
Source: Scand J Trauma Resusc Emerg Med. 2021 Mar 8;29:44. doi: 10.1186/s13049-021-00858-6 (PMC7938460; doi:10.1186/s13049-021-00858-6)

Additional file 3. Funnel plot for favorable neurological outcome at hospital discharge, according to mixed-effects meta-regression model


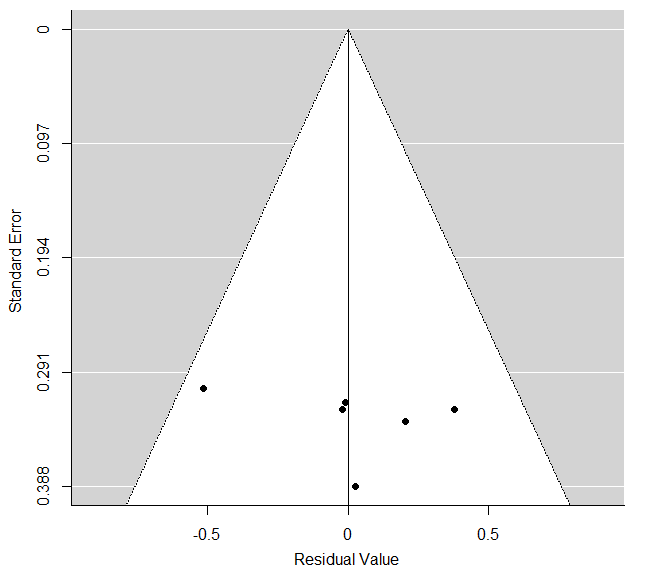

Supplement: Supplementary file 3 — Additional file 3. Funnel plot for favorable neurological outcome at hospital discharge, according to mixed-effects meta-regression model. [file 13049_2021_858_MOESM3_ESM.docx]
